# Serious Games for Executive Functions Training for Adults with Intellectual Disability: Overview

**DOI:** 10.3390/ijerph191811369

**Published:** 2022-09-09

**Authors:** S. Shapoval, Mercé Gimeno-Santos, Amaia Mendez Zorrilla, Begoña Garcia-Zapirain, Myriam Guerra-Balic, Sara Signo-Miguel, Olga Bruna-Rabassa

**Affiliations:** 1 eVIDA—Lab, Deusto University, Avda/Universidades 24, 48007 Bilbao, Spain; 2 Faculty of Psychology, Education and Sport Sciences, Blanquerna, University Ramon Llull, C/Císter, 34, 08022 Barcelona, Spain

**Keywords:** intellectual disability, adults, executive functions, cognitive functions, serious game, mobile app, game training

## Abstract

(1) Background: Throughout the history of medical and psychology practice, specialists have worked to improve the quality of treatment and rehabilitation, which has led to the emergence of concepts such as serious games. These tools focus on different areas of intervention procedures, one of which is to help people with intellectual disability (ID). Individuals with ID have problems with executive functions (EFs), which are related to adaptive functioning. Recent studies showed that serious games positively impact cognitive, social, and communication skills in people with ID. The purpose of this study is to analyze the solutions that have been found in EF training for adults with ID in recent years, evaluating them with a number of key parameters and identifying the features and possible problems in the further development of our system. (2) Methods: A review was conducted starting with 573 articles in English related to serious games and selected from studies that had been published since 2015. Finally, 10 were examined in detail as they focused on EFs in adults with ID. They were searched in seven major databases (“Association for Computing Machinery” (ACM), IEEE Xplore database, DBLP computer science bibliography, Google Scholar, PubMed, SCOPUS, and PsycInfo). (3) Results: It was determined that the most frequent EFs referred to in the studies analyzed were planning and decision-making, followed by working memory and social cognition, behavioral regulation, flexibility, and inhibition capacity. The basic approach to the creation of support systems was also analyzed in terms of technical and program execution. The trend results’ analysis evidenced improvements in EFs, even though they were not significant. This comprehensive technique enabled the identification of the main features and aspects to be taken into account for further development of our system.

## 1. Introduction

According to the American Association on Intellectual and Developmental Disability [1], intellectual disability (ID) is a disability characterized by limitations in intellectual functioning and adaptive behavior, which appear before 22 years of age, considered as the developmental period. Intellectual functioning is understood as a mental capacity for learning, reasoning, or problem-solving, among other aspects Adaptive behavior includes conceptual, social, and practical skills that enable people to adequately perform daily living skills (eating, dressing, cleaning, etc.), occupational tasks, self-care and healthy living, agenda management, money use ability, transportation use ability, and other aptitudes [1]. Finally, it involves having the ability to cope with life as an independent individual.

The AAIDD also considers other factors that may determine disability, for example the community and language or cultural issues. In any case, these persons might function better if they receive personalized support.

ID appears when the development of the brain is disrupted before or during birth, as well as during childhood. One of the most frequent causes of ID is Down syndrome (DS). It is associated with some physical features that give a characteristic appearance [2].

It is remarkable that improved medical care has increased the life expectancy of persons with ID, including DS, which therefore calls for the promotion of a good quality lifestyle [3,4,5,6]. This is a challenge when considering the developmental cognitive impairments associated with lifelong intellectual disability and, moreover, the variability of cognitive functioning across individuals [7]. With greater survival rates reported among people with ID, there has been an increase in physical, cognitive, and mental health challenges [8].

The executive functions are key elements in human development as they comprise a multidimensional construct that includes response inhibition, working memory, and cognitive flexibility [9]. Moreover, Reference [10] indicated that working memory, shifting, and inhibition capacity facilitate other EFs such as planning and problem-solving. Among the general population, good executive function performance, and in particular self-regulatory capacity, is linked to the ability to suppress inappropriate behavior and seek conduct that is better adapted. A good result in executive functions in the early life stages encourages healthy social relationships in adulthood, better levels of employment, and a lower response to risky behavior [11,12]. Together with other cognitive functions, response inhibition is a process that aims to promote cognitive performance adjusted to one’s environment, and this is a key component in self-regulation processes [13,14].

There is a growing body of literature that explores the nature of executive functioning in individuals with ID [15]. However, the research is inconsistent regarding the relationship between executive functioning and general intellectual ability [16]. The relationship found may depend on the population studied and the measurements used to examine executive functioning and intellectual ability. There is considerable evidence pointing to the decline of executive functioning in the aging process [17]. Individuals with ID may show levels of executive functioning commensurate with their developmental level [18]. Recent studies suggest that individuals with ID have difficulties with EFs, albeit there are differences in assessment measures. Better understanding of these characteristics and validated tests for this population will thus aid in assessing the effectiveness of interventions [19].

A positive correlation has been found between executive and adaptive functioning in individuals with ID [20]. Deficits in executive functioning have been linked to challenging and offending behavior [21]. Adults with ID may be more at risk of developing more severe dementia than expected in old age, especially those with DS [22,23,24]. Therefore, early detection of dementia can be challenging in persons with ID [25]. It has also been suggested that people with DS manifest executive dysfunction and behavioral and psychological symptoms in the pre-clinical stages of Alzheimer’s disease (AD) that may precede loss of memory [26]. It has been pointed out that one indicator of AD in persons with DS is the loss of daily living skills [27], which makes boosting their cognitive reserve vital. Taking into account the changes in executive functions in the adult population with DS is therefore extremely important to assess the decline of such functions in people with DS, as it could contribute to early diagnosis of AD onset and treatment to promote a better quality of life [28]. Moreover, recent studies showed significant improvement of computerized cognitive training on levels of executive function in adults with DS [29].

The most common type of game in the literature is serious games, but the studies are more focused on the development of cognitive abilities rather than of adaptive skills, especially in the child population with ID [30,31]. A positive impact of serious games for people with ID or autism spectrum disorder has been shown, in relation to cognitive, social, and communication skills [32]. The use of new technologies facilitates interactive learning, which allows reinforcing the learning carried out through the different platforms and helps to develop new learning. Among them is the use of serious games. These solutions help in the training and adaptation of people with ID, as well as allow for more accurate monitoring of their condition. Using such systems, it is possible to teach people who require assistance in adapting to society due to different circumstances using the most accurate examples, but most importantly, they allow them to monitor their performance remotely. 

Previous reviews related to serious games for people with ID analyzed how to design them to adapt to their cognitive requirements, to examine their general effects on people with ID or autism, to evaluate their adaptation for inclusive learning and teaching in this population, or to study their accessibility, considering cognitive, motor, and sensory disabilities [33,34,35], but not to improve their executive functions.

This review seeks to analyze serious games designed to help and support adults with ID for executive functions training, considering that these functions are relevant for adapting to daily living activities and to promote autonomy. We focus on serious games for adults with ID because there are already many kinds of solutions created specifically for children and adolescents, and the purpose is to analyze variants of such programs or applications, to understand their main features, and based on the received data, to draw conclusions about the effectiveness of serious games [36,37,38,39,40].

During the analysis, a few fundamental questions also need to be answered. In the long run, they will help better understand the essence of the topic under study, as well as to determine further research plans on game solutions for people with intellectual disabilities. The work raises four main issues, which are described in Table 1.

## 2. Materials and Methods

### 2.1. Methodology

In order to qualitatively evaluate existing solutions in terms of serious games for people with ID, it is crucial to choose the right criteria for finding relevant materials. For the search process, information sources must meet clearly elaborated and defined basic and technical and semantic criteria to be acceptable. 

Additional reading on the criteria is required. First of all, the scientific work or article must be technical in nature, containing a technical and experimental description of the proposed solution, whether it is a mobile application or another user-focused engineering product. It is also important to describe the target audience for which the solution is intended. 

To search for such articles, a set of keywords was compiled, which were unique tags such as Game, Disability, Mobile, Intellectual, and so on. A combination of them was selected to narrow down the search and make it possible to find the source more accurately. A total of 11 such combinations were used, all of which are shown in Table 2.

### 2.2. Databases

The search was performed in the following 7 databases: Association for Computing Machinery (ACM), IEEE Xplore database, DBLP computer science bibliography, Google Scholar, PubMed, SCOPUS, and PsycInfo. The results of the search are shown in Table 2, which also shows the whole number of articles that included one or a combination of several keywords.

### 2.3. Inclusion and Exclusion Criteria

**Inclusion criteria:**

- Population: adults (18+) with ID.
- Intervention: Digital solutions based on serious games, acting as support, and learning assistants. Applications for mobile platforms and personal computers (PC).
- Publication source: Journals included in the following databases: “Association for Computing Machinery” (ACM), IEEE Xplore database, DBLP computer science bibliography, Google Scholar, PubMed, SCOPUS, and PsycInfo.
- Time period: Papers published since 2015.
- Language: English.

**Exclusion criteria:**

- Population: (1) The diagnosis of an ID was not explicitly mentioned; (2) people diagnosed with mental illness, acquired cognitive or neurological impairments, blindness, or physical disabilities.
- Kind of document: Conference proceedings/abstracts, editorials, dissertations/theses, or published in non-peer-reviewed journals.
- Language: A full text was not available in English.
- Intervention: Applications for entertainment, leisure, or other entertaining events.

It is worth determining for what reason in the inclusion criteria the year parameter to search for articles was defined as 2015 or newer. Such a time interval of 5–7 was chosen due to the fact that about this period is necessary for the development and implementation of new technologies in the creation of computer games and similar systems. 

Furthermore, a comparison of the number of results for any of the keywords suggested by the authors for a similar time period, for example, 2009–2015, shows that, since 2015, the amount of research and development in the direction of game assistants has rather increased.

In addition, this parameter of the selection and filtering of articles was chosen based on the direct chronology of the development of the computer technology and gaming industry. In this case, several classifications are applicable, such as, for example, by graphics technology or applicable benchmarks. In the case of this particular study, the authors were interested in control technologies and player feedback.

This should be understood as follows. If you take the entire chronology of computer games (it does not matter if they are entertainment applications, helper applications, monitors, and so on), you can distinguish several stages of the methods that were used for the interaction of the player with the system. Beginning in the 1960s, there were primitive algorithms that were controlled, for example, by the same controllers that were responsible for adjusting the sound on the machine. Then, beginning in the late 1970s, the first arcade machines began to appear, which were a screen, a controller, and a game at the same time. The 1990s gave us game consoles. The early 2000s saw the rise of the personal computer as a gaming platform, making the keyboard and mouse the “canonical means of control”, followed in the 2010s by the Kinect, Wii U, and a number of others, which allowed one to interact with games through movement.

Directly, these systems have already allowed in 2014–2015 moving to modern implementations of virtual reality; although the development of these systems was carried out before, the capacity of the equipment did not allow bringing the technology to an acceptable form. Parallel to this, again due to the increase in the capabilities of computer equipment, developments began in the application of machine learning algorithms and neural networks in motion tracking and the implementation of these systems as assistants in various areas of industry. As at the moment, these systems are actively evolving in terms of accuracy, performance, and capabilities, this study looked at developments that were made just in this period of development.

It is also important to clarify the point of choosing a particular branch of gaming applications, namely serious games. As one of the most common definitions states, serious games or applied games are applications designed for a primary purpose other than pure entertainment. The “serious” adjective is generally prepended to refer to video games used by industries such as defense, education, scientific exploration, health care, emergency management, city planning, engineering, and politics. The main purpose of such applications is to educate, assist, or support users in various situations.

Given that there are many such areas, the authors of this article considered a particular branch, namely systems aimed at assisting and monitoring the elderly, as well as people with intellectual disabilities. The applications and systems chosen subsequently also met certain categories, namely:

Exercise therapy: The systems being developed are considered primarily as a supplementary tool for the rehabilitation and support of people with ID. In essence, they are intended to be analogous to, or complementary to, exercise. 

Adult and youth education: In this case, the systems in question should be able to be used as learning, teaching, or organizing systems. This applies to the elderly, as well as to people with special needs. This includes help systems for activities of daily living, organizers, navigation applications, and assistants with all kinds of instructions. 

Health: In this case, systems are considered that include the possibility of reusable and long-term use.

### 2.4. Flow Chart

**Figure 1:**
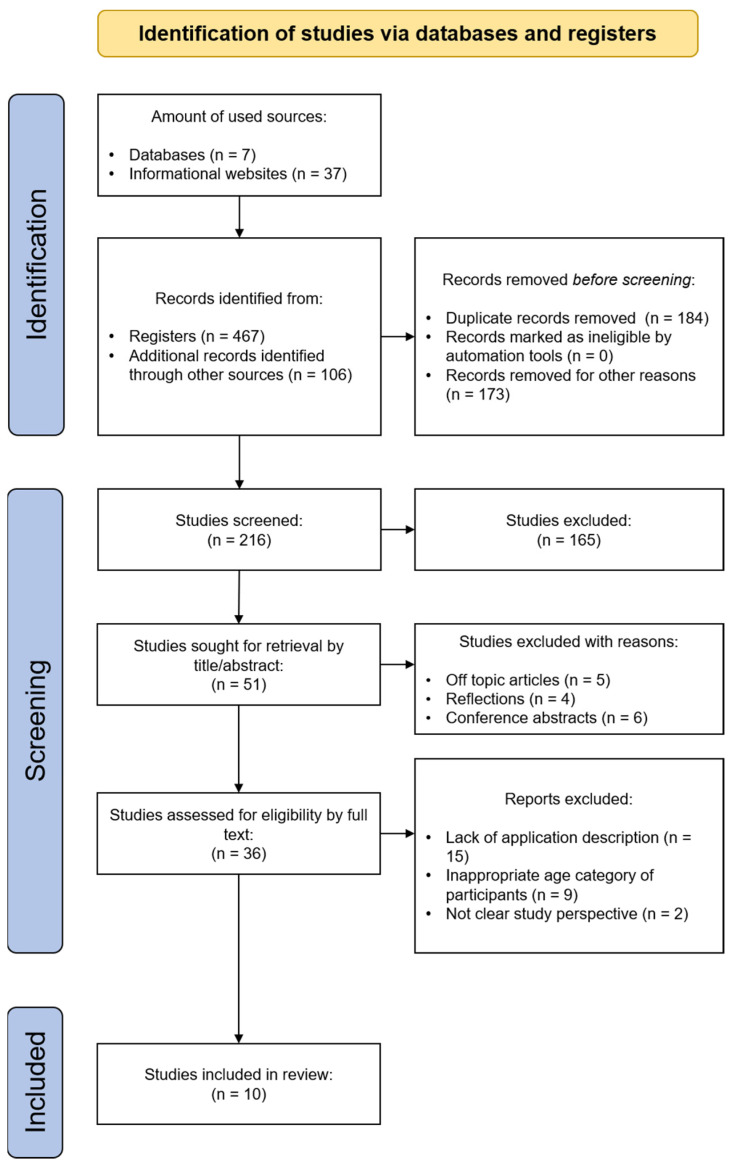
Flow chart.

Figure 1 shows the flow chart of the article search and filtering process. The authors selected and filtered 573 articles, finally selecting 10. The evaluation and filtering were carried out by two researchers on each article. If no agreement was reached, a third researcher evaluated the paper.

A keyword search of the articles in Table 3 selected 573 entries. All the duplicates or nearly identical articles were then removed, yielding a document set consisting of 357 texts. Then, 216 iterations were run on all the articles, resulting in 167 samples being excluded from the set of suitable ones due to the presence of studies published in non-scientific sources, an unclear structure, a difference from the desired focus (articles that describe gaming or functional applications), conference proceedings/abstracts, editorials, dissertations/theses, or published in non-peer-reviewed journals. The result of this filtering was a list of 36 studies that met the original work requirements as closely as possible. Ultimately, after additional filtering and primary analysis of articles that met all of the criteria described above, the top 10 articles in the set were taken for further analysis.

### 2.5. Additional Quality Evaluation

Additional evaluation criteria to assess the quality of the review had to be defined for each of the articles selected. There were 12 such criteria, and they are described in Table 3, which explains additional criteria that more accurately state the content of each of the selected studies.

Each of the evaluation criteria was given a weight parameter, which corresponds to the importance and overall role in shaping the article. Table 4 directly shows the evaluation characteristics of the articles. Most of the criteria followed the PRISMA recommendations for reviews, and the authors added items ad hoc.

It is also worth mentioning the item Innovation, which was added to the previous PRISMA criteria. This parameter was evaluated based on several criteria: the technology that was used to create the application, the basic algorithms used to create the system (neural networks, artificial intelligence, conventional software solutions, etc.), as well as taking into account the uniqueness and functionality of the application. The more comprehensive and functional in terms of technology an application is, the higher the grade level on a scale of 1 to 3. This characteristic was introduced in order to understand how different degrees of “manufacturability” affect the result of the real-world use of such systems, which will help to grasp what tools are needed and how our own application should be developed in the future. Overall quality, in turn, was determined by the sum of the parameters described in Table 3 and in the same way corresponds to the importance and overall impact on the impact value of each article.

## 3. Results

The search and processing of the articles produced basic tables that summarize the main points of each of the studies selected. The analysis of the selected articles including the most important criteria is presented in Table 5. The information given in them characterizes the applications presented in each specific study.

In Table 6, we included the different components of executive functions as Planning, Working Memory, Flexibility, Inhibition, Behavior Regulation, Social Cognition, and Decision-Making. Moreover, we also included another column related to other cognitive functions included in the studies, taking into account that they are related to EFs and in order to provide additional information.

**Figure 2:**
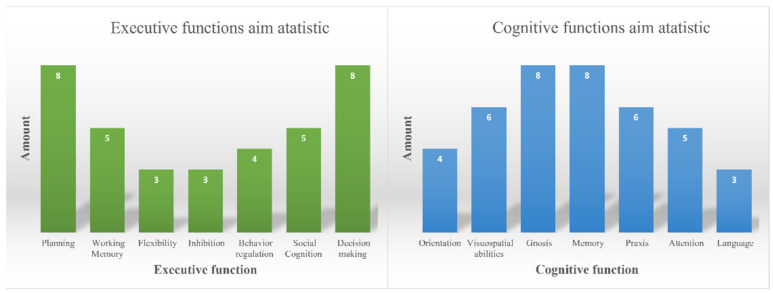
Statistics on the focus of research in relation to the main executive and cognitive functions.

Based on the results of Table 6, it is possible to assess which functions the researchers emphasized most often. This information is shown in Figure 2. 

Figure 2 illustrates well which functions the authors of the articles paid most attention to. Thus, among executive functions, the most emphasis is placed on Planning and Decision-Making, and among cognitive functions—Gnosis and Memory.

Table 6 shows the result and structure of the primary analysis of the items used. Thanks to this stage of analysis and the additional evaluation criteria introduced, it was possible to identify the most relevant articles, which became the basis for the study presented. More in depth information is presented in the Results Section. In Table 7, we included demographics, type and level of ID, objectives, designs, results, and outcomes related to the serious games studies selected.

The articles selected for this study in the sorting process had to include one of the most important parameters, namely the presence and description of a developed and tested application from the serious games category. Based on the data presented in the studies used, an analysis of these applications was performed, and both a technical and functional report on the solutions, methodologies, and goals pursued by the authors was compiled. 

During this analysis, it was important not only to understand the technical part, namely in what year the study was carried out, the anticipated platform, technical characteristics, and so on, but above all, the condition and characteristics of the target audience. Table 7 presents the criteria according to which the authors of this article will conduct future research and develop their own solutions.

**Figure 3:**
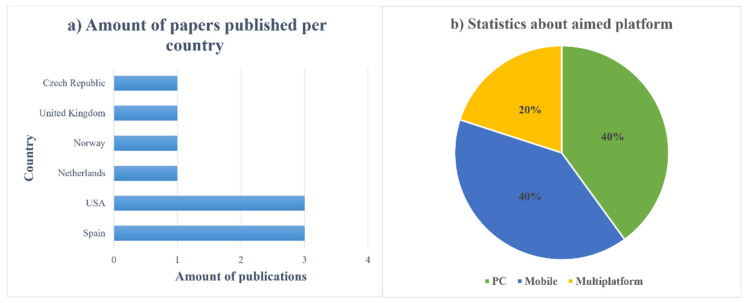
Results of the review of Table 6 and Table 7: (**a**) amount of papers published per country; (**b**) aimed platform statistics.

Figure 3b shows the statistics on the percentage of device platforms where the selected articles were found. Based on the information received, an equal number of platforms hit personal computers and mobile devices. Twenty percent was allocated to multi-platform use.

**Figure 4:**
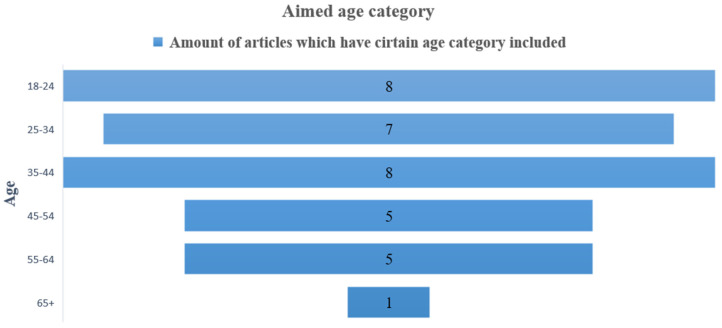
Aimed age category.

Figure 3 and Figure 4 also illustrate the main points of the analysis of the items in Table 6 and Table 7, respectively, including the age group of participants considered in the selected studies. In Figure 4, it can be observed that the age group that has been most studied is the one corresponding to young adults (18–44 years old). The numbers show how many articles include a certain age group.

## 4. Discussion and Conclusions

This review analyzed serious games or mobile apps that have been designed for adults with ID. Those created for children and adolescents were rejected taking into account that this age group with ID is usually more familiar with new technologies, and there are many kinds of apps specifically for them. Instead, serious games that focus on adults with ID are few, and they need to develop executive functions to facilitate daily life during their aging process. It is also important to have good support and a learning process to maintain their cognitive reserves as they grow older.

The analysis of the selected articles enabled us to draw certain conclusions about the general situation of the development of tools to support and train people with ID.

It is worth analyzing the time of publication of articles. As can be seen in Table 6, research on this topic peaked in 2019. This trend applies to four articles with high and medium impact factors. However, the overall situation has remained stable since 2015 in terms of the number of publications on the topic of serious games for people with ID. It is also interesting to note the fact that, after 2019, the number of studies fell to the level of 2016–2018. This could perhaps be related to the COVID 2019 pandemic due to difficulties in development and research, as well as testing with the target audience.

It is also interesting to consider which countries developed these studies. Spain and the United States have published most of the articles, three respectively, as shown in Table 6. However, the number of such studies in other countries is much lower. This may be due to various factors, such as the direction of social policy and the level of development of technology, medicine, and general attitudes in society. 

Concerning the age of the intended audience, given the characteristics of the people targeted in this study and the articles we selected, it is worth taking a comprehensive approach and including many factors. Our research focused on people with ID, so it is necessary to thoroughly study their characteristics to know the level of ID, their lifestyle, the quality of their education in the early life stages, etc. This will determine not only the level and degree of support needed with apps, but also the structure of such systems. It is at this stage that certain limitations arise, which will affect further research and development of future apps. 

However, many articles found were related to a population of children [31,32,33,34]. The information provided in these articles is important at least as an example of the app development and its application for children. Especially in the early stages of learning, the mental development of people with ID is often comparable to that of preschool- and elementary-school-age children. Therefore, the information on techniques in these articles is relevant, despite the marked age limitations of the audience.

We include the discussion of the findings with respect to the defined research questions proposed in the study.

**Q1—What are the characteristics of the participants in the studies?**

In relation to the sample size of the selected articles, they ranged from 3 to 172 participants. The sample included in five of the papers was over 30 participants, while 5 of them had fewer participants, taking into account that two of the articles were related to the same study sample [41, 51]. 

In relation to the age criteria, the new concept of ID considers that it appears before 22 years old [1]. Nevertheless, this new definition begun in January 2021, so we have included samples older than 18 years old, both because mainly all the previous papers published considered this age and because the worst moment during the COVID-19 pandemic was in 2020, when many serious games were developed, including people older than 18 years old. Three of the 10 papers that met the criteria were published in 2020, and the rest of the papers were published before.

Regarding the age of the participants, there was wide variability between studies, with a range between 18 and 62 years old, taking into account that there is considerable evidence pointing to the decline of executive functioning in the aging process [17,18,28]. The age range in the reviewed studies varied from 18 to 62 years old. Unfortunately, some studies included a sample of adolescents and adults without separating the age groups. Nevertheless, it is interesting to note that Reference [43], with a group of persons with Down syndrome from 15 to 36 years old, showed how they designed the Emotion4Down video game, which is a serious game that supports the emotional awareness in this population. This was also the case of [46], which included persons aged between 16 and 60 years. Nevertheless, these authors analyzed the effectiveness of an app for improving motivation for physical activity and, through it, psychological health, self-efficacy in activities, and social support. Self-efficacy can facilitate certain autonomy in the target population, and motivation is a good indicator that might also condition EFs. Nevertheless, these studies did not follow the inclusion criteria for the present review, although they did provide interesting information. 

Regarding gender, four articles indicated the gender of the participants and only two studies included a balanced sample of men and women [47, 52], while the remaining four articles did not identify the gender of the participants in the study.

We analyzed the articles that indicated that the serious game targeted people with ID, and we excluded those studies with participants diagnosed with mental illness, acquired cognitive or neurological impairments, blindness, or physical disabilities. It must be taken into account that an article was included in which a sample of five people with DS was analyzed, and it also included five people with perinatal hypoxia–ischemia [44]. Four of the studies included participants with DS, and the rest of participants had diverse etiologies (autism spectrum disorder, William syndrome, attention deficit hyperactivity disorder, and atypical behaviors of pervasive developmental disorder).

Regarding the level of disability, the studies included a range of ID that varied from mild, moderate, to severe.

**Q2—Which executive functions does the study mainly focus on?**

The second important factor is the executive functions that the study focused on. The analysis of the included studies was based on the dimensions of the EFs proposed in the previous literature, which include Inhibition, Working Memory, Cognitive Flexibility, Planning, and Problem-Solving [9,10,21]. In addition to these executive functions, Behavioral Regulation, Social Cognition, and Decision-Making were also included, taking into account that they are very important to self-regulation in daily life activities. Moreover, we considered it interesting to include other cognitive functions such as Orientation, Visuospatial Abilities, Gnosis, Memory, Praxis, Attention, and Language, in order to carry out a more complete analysis of the studies. Thus, the serious games analyzed help to improve EFs and cognitive functions during daily life activities, facilitating autonomy in people with ID. 

The most frequently mentioned EFs in the analyzed studies were Planning and Decision-Making, followed by Working Memory and Social Cognition, Behavioral Regulation, Flexibility, and Inhibition Capacity. It should be noted that the studies by [41, 51] were the ones that included all EFs, as well as other cognitive functions (Table 6). 

Planning and Decision-Making are more relevant for adaptation to daily living activities and promote autonomy in the general population during the aging process, but also in persons with ID. Working memory and social cognition are included in various studies, which are also relevant for adapting and promoting interaction with the environment. 

On the other hand, we want to emphasize that many of the serious games analyzed did not include Inhibition Capacity. We believe that it would be interesting to include this executive capacity, as some studies have indicated that Inhibition capacity is a predictive function of cognitive performance in people with ID [9,14].

Regarding cognitive functions, the most relevant are Memory, Gnosis, and Praxis, which are also related to EFs. We have to take into account that these functions are also very important to autonomy in daily living skills [26], and boosting the cognitive reserve is vital in the aging process of people with ID in order to promote quality of life [28,29].

Of the selected articles, there were no specific studies on EFs, as they were included with other cognitive functions. It would be interesting to design an application focused on EFs in order to promote the autonomy of people with ID.

**Q3—Do serious games have a positive impact on the EFs of participants?**

First of all, we had to consider that many of the studies on serious games have been carried out with children and research tends to be more focused on the development of cognitive abilities, while few have been conducted specifically in relation to EFs in ID [31,32,33,34,35]. 

Moreover, most of the reviewed studies did not include a precise evaluation of the EFs or the cognitive functions that are included in the serious game when assessing the effectiveness of the intervention. However, most of the serious games have been created with the objective of training cognitive functions, and the revied studies indicated that most of the serious games had positive effect on the improvement of independent living and promoting interaction with the environment in users with ID. This is consistent with the previous literature, which shows a positive impact of serious games on people with ID, in relation to cognitive, social, and communication skills [30].

Recent studies suggest that there are differences in assessment measures between research works regarding EF difficulties in persons with ID. Thus, better understanding of the EF characteristics and validated tests for this population will aid in assessing the effectiveness of interventions [21,22].

To adapt to daily life and improve autonomy, Planning and Decision-Making were the most frequently studied EFs. Working Memory and Social Cognition were included in various studies, which are also relevant for adapting and promoting interaction with the environment. It should be noted that the studies by [41, 51] were the ones that included all EFs, as well as other cognitive functions. The results indicated that there were differences between high-functioning and medium-functioning users and that almost all of the participants at least completed the task without mistakes. The general improvement through sessions and the low mistake ratio were good indicators of the appropriateness of the game design.

Most of the studies analyzed included EFs and other cognitive functions, such as Memory, Gnosis, and Praxis, which are also related to EFs and are relevant to promoting autonomy in daily living skills [26] and quality of life [28,29]. No specific EF was found in the articles studied, and cognitive functions were also included in some.

**Q4—What is the most promising platform for developing target applications?**

It is important to identify the most common platform for development. Regarding the platform for which the solutions were developed, the main objective of this study was to understand which platform is more popular and, therefore, in the long term, more effective in terms of both the development and training of people with intellectual disorders. Based on the analysis of the selected articles, which is shown in Figure 2, we can see that the situation is as follows: If we take the three variants of the distribution of solutions, namely personal computers, mobile platforms, meaning Android- and iOS-based systems, as well as solutions that can be run on all devices, such as web services, we see a roughly equal distribution: 40% were computer platforms; 40% were mobile platforms; 20% were multiple platforms. 

Despite the prevalence of PC platforms, thanks to the distribution of multiplatform solutions between PCs and mobile devices, an equal distribution of systems can be observed. This can be seen more clearly in Figure 3b. A study of high-impact-factor articles shows an equal balance of platforms. However, the interesting fact is that if we look closely at Table 5, we can see that research for use on mobile platforms has become increasingly popular since 2018, which is due to the development of technology for this type of personal device. Nevertheless, according to general source reviews, the most “common” and most expected platform is still PC. The same tendency could be seen among the 10 selected articles (Q3).

***Systems’ limitations for individuals with ID***

One of the first limitations is age. Initially, it was decided to set the age bar starting at 16 and ending at 70–75 years old. This was justified by several reasons. First, children under 16, especially those with such disabilities, are usually fully cared for and supported by their parents, in some cases by close relatives, or by competent authorities and services. In contrast, by the age of 16, a person must already know how to function in society and prepare for what is called “adult independent life”. In this sense, that is the age at which the tools presented in this study should begin to be used. As for the upper threshold of 70–75 years, there are also peculiarities. Despite the fact that people with Down syndrome present a shorter life expectancy and although modern medicine and therapy methods allow extending this to 50 years and even longer, the different intellectual levels of this syndrome and comorbidities may render these systems semi-invalid in different daily life situations. Furthermore, this kind of learning system may also be useful for elderly people with intellectual disabilities. It is also possible to teach older people some activities or systems that are difficult to understand or comprehend due to their age.

The second limitation would simply be the factor of human learning. Again, given the peculiarities of the disease under study, the person using the system must be at least basically be able to learn. Otherwise, each launch of the application would seem like the first time, which would be useless, as the purpose of such systems will be to teach them how to function and exist in society.

Another important limitation is the fact that the additional tools, such as the apps described in this study, must be used in the presence of a mentor/teacher/doctor. In such systems and applications, in addition to the main function of supporting users, another most important issue is the system of collecting information on their success and progress. This is necessary to more accurately monitor the progress of learning, as well as to understand the overall state of the individual.

Such an app can also provide much more information than the supervisor can obtain or even more than can be shared by the user. In addition, the tutor will help to understand the system more accurately, and if necessary, to direct or further explain it in addition to the task or activity.

Having analyzed in detail the information we received, we can determine how appropriate and promising it is to conduct further work in this direction. Based on the selected 10 articles and after analyzing all their features, we concluded that continuing research in this direction is promising for a number of reasons. The popularity of such support systems is currently growing. This is due both to the improvement of technology in physical terms, as well as improving and simplifying methods of creating software for all areas in general, and specifically in order to promote autonomy. Another important factor is that, unlike traditional methods, this system allows one to obtain more information about the user’s performance, without any additional tools.

Based on the data obtained in this study, further development of such a system will be aimed at creating its own platform to help people with ID to adapt to social life. The development is planned in the direction of the mobile device market and will involve both educational functions and training systems based on mobile 2D and 3D games.

The way in which the applied system interacts with the participant also deserves special attention. The manner in which the purpose of the activity is explained, its features, and the way it is performed are vital. Based on this information, it is possible to judge the inclusiveness of the solutions presented and their focus and user-friendliness parameters

In general, all the proposed articles, as well as any application of this kind, can be divided into two types: for independent use and with the participation of a supervisor. The former, in turn, are divided into the use of explanatory instructions, pop-ups, or intuitive use, in which the users themselves should guess the goal and the way to perform the task. On the one hand, this method is very effective in terms of developing self-organization and self-learning, as well as improving skills. However, unlike assistance from a mentor, users of self-study methods are more prone to erroneous learning methods. Moreover, this method is worth using in cases in which the patient’s condition is considered less critical, although it is not appropriate for severe cases of intellectual disability.

Regarding solutions in which the main role is played by the tutor, this arrangement is very good in the initial stages of training, as a specialist can control the process and minimize the initial error in the user’s progress. In cases where the mentor method is used for too long, a moment of stagnant progress can occur, thereby negatively affecting the user. On the other hand, unlike the first type (independent use), this method expands the range of degrees and levels of intellectual disabilities to which it can be applied, and if used incorrectly, it can cause a loss of personal independence and greater dependence on the decisions and presence of a mentor.

There is also a third type of application, which is essentially a mixture of the previous two. Incorporating the strengths of the first and the second type, it also balances their weaknesses. It is more universal, but more difficult to implement. In addition, due to the higher level of activity content, it can be used for a larger set of special needs, without requiring refinement or customization for a specific case of the disease. 

## Figures and Tables

**Table 1:** Key research questions for the study presented.

Research Questions	Expected Answer or Purpose
Q1—What are the characteristics of the participants in the studies?	To know the type and severity of the population with ID that is addressed in these serious games
Q2—Which executive functions does the study mainly focus on?	Analysis of priority target executive functions
Q3—Do serious games have a positive impact on the EFs of participants?	Analysis of outcome results on executive functions
Q4—What is the most common platform for developing target applications?	Identifying the most common platform for development

**Table 2:** Database search results.

Keyword	Database						
	ACM	IEEE	DBLP	Scholar	PubMed	SCOPUS	PsycInfo
Intellectual Disability	18005	111	21	184000	21127	20655	63
Cognitive	24113	26220	7164	1140000	270843	343373	22
Game	35751	27217	11409	1060000	15650	134404	0
Serious Game	48082	2417	621	454000	947	7780	0
Mobile App	63687	5422	3416	281000	14196	19069	73
Executive Function	24686	60	70	1170000	21029	32104	809
(Intellectual disability) AND (Game)	48991	33	4	21000	35	275	1
(Cognitive) AND (Serious Game)	62507	268	19	72100	211	982	2
(Intellectual disability) AND (Mobile App)	73440	6	1	10700	31	37	0
(Executive Function) AND (Serious games)	25463	12	1	17000	30	452	799
(Mobile App) AND (Intellectual Disability)	10405	7	2	16100	42	18	67

**Table 3:** List of criteria for the analysis of articles’ quality [36].

	Item Number	Description	Value	Weight
About the text of the article itself (5 points)	1	Provide in the abstract an informative and balanced summary of what was performed and what was found	1, 2, 3 (bad disc, average quality, good quality)	1
	2	Give the eligibility criteria and the sources and methods of the selection of participants	1, 2, 3 (less than 10, more than 10, more than 50, 0 if no information)	
	3	Discuss limitations of the study, considering sources of potential bias or imprecision	1/0 (were they in the study or not, 0—was, 1—was not)	
	4	Cautious overall interpretation of results considering objectives, limitations, and a multiplicity of analyses	1, 2, 3 (bad disc, average quality, good quality)	1
	5	Results from similar studies and other relevant evidence	1/0 (new solution/based on existing one)	0.5
Other quality metrics (5 points)	6	Was the study or method based on an existing solution?	1/0 (1—public/0—private)	2
	7	Was the dataset used in the research public or private?	1/0 (yes/no)	1
	8	Is the application public?	1-2-3	0.5
	9	Innovation?	1/0 (yes/no)	0.5
	10	Do testers show better results using the solution presented?	1-5 (depends on year and citations)	1
Additional (2 points)	11	Number of citations/years?	1, 2, 3 (PC, mobile, multiplatform)	0.5
	12	Platform	1—journal, 0—conference	0.5
Quality criteria		Journal/conference	High	
		18 or higher	Medium	
		14–17	Low	

**Table 4:** Quality analysis of selected articles including executive and cognitive functions.

	Article/Reference	Item												Result	Impact Factor Value
		About the Text of the Article Itself					Other Quality Metrics					Extra			
		1	2	3	4	5	6	7	8	9	10	11	12		
1	**McMahon et al. (2015)** *Effects of Digital Navigation Aids on Adults With Intellectual Disabilities: Comparison of Paper Map, Google Maps, and Augmented Reality* [41]	**1**	**1**	**1**	**2**	**1**	**0**	**0**	**2**	**1**	**2**	**2**	**1**	**12.5**	***Low***
2	**Peñaloza-Salazar et al. (2016)** *Cognitive mechanisms underlying Armoni: A computer-assisted cognitive training programme for individuals with intellectual disabilities* [42]	**3**	**3**	**1**	**3**	**1**	**0**	**0**	**2**	**1**	**2**	**1**	**1**	**18**	***High***
3	**Cano, A.R., Fernández-Manjón, B. and García-Tejedor, Á.J. (2018)** *Using game learning analytics for validating the design of a learning game for adults with intellectual disabilities* [43]	**2**	**3**	**1**	**3**	**1**	**1**	**0**	**2**	**1**	**4**	**3**	**1**	**19.5**	***High***
4	**Cano, García-Tejedor, Alonso-Fernández, and Fernández-Manjón, B. (2019).** *Game Analytics Evidence-Based Evaluation of a Learning Game for Intellectual Disabled Users* [44]	**2**	**3**	**1**	**3**	**1**	**0**	**0**	**2**	**1**	**3**	**1**	**1**	**17.5**	***Medium***
5	**Derks et al. (2019)** *Effectiveness of the serious game ‘You & I’ in changing mentalizing abilities of adults with mild to borderline intellectual disabilities: a parallel superiority randomized controlled trial* [45]	**2**	**3**	**1**	**3**	**1**	**1**	**0**	**3**	**1**	**4**	**1**	**1**	**19.5**	***High***
6	**Lara et al. (2019)** *A Serious Videogame to Support Emotional Awareness of people with Down Syndrome* [46]	**2**	**2**	**1**	**3**	**1**	**0**	**0**	**2**	**1**	**3**	**1**	**1**	**16.5**	***Medium***
7	**Benda et al. (2019)** *Practical Education Of Adults With Intellectual Disabilities Using A Web Course* [47]	**2**	**2**	**0**	**3**	**1**	**0**	**0**	**2**	**1**	**3**	**1**	**1**	**16**	***Medium***
8	**Bridges et al. (2020)** *Augmented Reality: Teaching Daily Living Skills to Adults With Intellectual Disabilities* [48]	**2**	**2**	**0**	**2**	**0**	**0**	**0**	**2**	**1**	**5**	**3**	**1**	**15**	***Medium***
9	**Michalsen et al. (2020)** *Physical Activity With Tailored mHealth Support for Individuals With Intellectual Disabilities: Protocol for a Randomized Controlled Trial* [49]	**3**	**3**	**1**	**3**	**1**	**0**	**0**	**2**	**1**	**4**	**2**	**1**	**19.5**	***High***
10	**Gibson et al. (2020)** *Designing Clinical AAC Tablet Applications with Adults who have Mild Intellectual Disabilities* [50]	**2**	**1**	**0**	**2**	**1**	**0**	**0**	**2**	**1**	**4**	**2**	**1**	**14**	***Medium***

**Table 5:** Main review of studied applications.

Reference	Year [Since 2015]	Country	App/Software Used	Platform (Android, IOS, PC)	Oriented to (Daily Life Activities—Leisure, Job)	Description of Applications
**McMahon et al. (2015)** *Effects of Digital Navigation Aids on Adults With Intellectual Disabilities: Comparison of Paper Map, Google Maps, and Augmented Reality* [41]	2015	USA	No information	Mobile	Training	Augmented reality navigation application was functionally the most effective condition, in the context of supporting people with intellectual disorder by teaching navigation skills.
**Peñaloza-Salazar et al. (2016)** *Cognitive mechanisms underlying Armoni: A computer-assisted cognitive training programme for individuals with intellectual disabilities* [42]	2016	Spain	No information	PC	Education	Naming ability, visual memory, comprehension, and vasoconstriction contributed the most to the predictive models regarding performance on the Armoni activities.
**Cano, A.R., Fernández-Manjón, B. and García-Tejedor, Á.J. (2018)** *Using game learning analytics for validating the design of a learning game for adults with intellectual disabilities* [43]	2018	Spain	Based on Google Maps	Multiplatform	Training	Tendency for users to use the same character in all the sessions; we did not observe differences in the play patterns between the players that customized the character and those who did not.
**Cano, García-Tejedor, Alonso-Fernández & Fernández-Manjón, B. (2019).** *Game Analytics Evidence-Based Evaluation of a Learning Game for Intellectual Disabled Users* [44]	2019	Spain	No information	PC	Education	The article presents a version of a game system to teach people the basics of social aspects, namely the use of public transportation.
**Derks et al. (2019)** *Effectiveness of the serious game ‘You & I’ in changing mentalizing abilities of adults with mild to borderline intellectual disabilities: a parallel superiority randomized controlled trial* [45]	2019	Netherland	No information	PC	Learning	The serious game “You & I” aims to improve mentalizing abilities in adults with mild to borderline intellectual disabilities.
**Lara et al. (2019)** *A Serious Videogame to Support Emotional Awareness of people with Down Syndrome* [46]	2019	USA	Unity	Mobile	Training	The Emotion4Down video game, a serious videogame that supports the emotional awareness of people with Down syndrome.
**Benda et al. (2019)** *Practical Education Of Adults With Intellectual Disabilities Using A Web Course* [47]	2019	Czech Republic	No information	PC	Education	The presented research examined whether a web course could be an effective educational technology for people with intellectual disabilities focusing on the repetition of knowledge.
**Bridges et al. (2020)** *Augmented Reality: Teaching Daily Living Skills to Adults With Intellectual Disabilities* [48]	2020	USA	Unity	Multiplatform	Training	Results indicate the intervention was effective for increasing independence among all participants.
**Michalsen et al. (2020)** *Physical Activity With Tailored mHealth Support for Individuals With Intellectual Disabilities: Protocol for a Randomized Controlled Trial* [49]	2020	Norway	No information	Mobile platform	Learning	The results of the study will determine the effectiveness and sustainability of a tailored mHealth support intervention to increase PA in youth and adults with IDs.
**Gibson et al. (2020)** *Designing Clinical AAC Tablet Applications with Adults who have Mild Intellectual Disabilities* [50]	2020	United Kingdom	Unity	Mobile platform	Training	Has the potential to assist people with mild ID throughout all aspects of life.

**Table 6:** Selected studies including executive and cognitive functions.

	Article/Reference	Studding Functions	
		Executive Function	Cognitive Functions
1	**McMahon et al. (2015)** *Effects of Digital Navigation Aids on Adults With Intellectual Disabilities: Comparison of Paper Map, Google Maps, and Augmented Reality* [41]	Planning, Decision-Making	Orientation, Visuospatial Abilities
2	**Peñaloza-Salazar et al. (2016)** *Cognitive mechanisms underlying Armoni: A computer-assisted cognitive training programme for individuals with intellectual disabilities* [42]	Planning, Working Memory, Flexibility, Inhibition	Visuospatial abilities, Gnosis, Memory, Praxis, Attention, Language
3	**Cano, A.R., Fernández-Manjón, B. and García-Tejedor, Á.J. (2018)** *Using game learning analytics for validating the design of a learning game for adults with intellectual disabilities* [43]	Planning, Working Memory, Flexibility, Inhibition, Behavioral Regulation, Social Cognition, Decision-Making	Orientation, Visuospatial Abilities, Gnosis, Memory, Praxis, Attention, Language
4	**Cano, García-Tejedor, Alonso-Fernández and Fernández-Manjón, B. (2019).** *Game Analytics Evidence-Based Evaluation of a Learning Game for Intellectual Disabled Users* [44]	Planning, Working Memory, Flexibility, Inhibition, Behavioral Regulation, Social Cognition, Decision-Making	Orientation, Visuospatial Abilities, Gnosis, Memory, Praxis, Attention, Language
5	**Derks et al. (2019)** *Effectiveness of the serious game ‘You & I’ in changing mentalizing abilities of adults with mild to borderline intellectual disabilities: a parallel superiority randomized controlled trial* [45]	Social Cognition, Decision-Making	Memory, Gnosis, Praxis
6	**Lara et al. (2019)** *A Serious Videogame to Support Emotional Awareness of people with Down Syndrome* [46]	Social Cognition, Decision-Making	Attention, Memory, Gnosis
7	**Benda et al. (2019)** *Practical Education Of Adults With Intellectual Disabilities Using A Web Course* [47]	Planning, Working Memory, Decision-Making	Visuospatial Abilities, Gnosis, Memory, Praxis
8	**Bridges et al. (2020)** *Augmented Reality: Teaching Daily Living Skills to Adults With Intellectual Disabilities* [48]	Planning, Working Memory, Behavioral Regulation	Gnosis, Memory, Attention
9	**Michalsen et al. (2020)** *Physical Activity With Tailored mHealth Support for Individuals With Intellectual Disabilities: Protocol for a Randomized Controlled Trial* [49]	Planning, Behavioral Regulation, Decision-Making	Orientation, Visuospatial Abilities, Praxis
10	**Gibson et al. (2020)** *Designing Clinical AAC Tablet Applications with Adults who have Mild Intellectual Disabilities* [50]	Planning, Social Cognition, Decision-Making	Memory, Gnosis

**Table 7:** Demographics, type and level of ID, objectives, designs, results, and outcome about the serious games selected.

Reference	N	Age	Sex	Type ID	Level ID	Objectives	Design	Result/Conclusion
**McMahon et al. (2015)** *Effects of Digital Navigation Aids on Adults With Intellectual Disabilities: Comparison of Paper Map, Google Maps, and Augmented Reality* [41]	6	18–24	4 males and 2 females	Mild to moderate ID	IQ 48–62	Effects of digital navigation aids on adults with intellectual disabilities	Teaching young adults with ID to (a) access the necessary technology, (b) apply the knowledge needed to use the tool or app, (c) make a decision based on the information obtained, and (d) utilize embedded digital supports; learners with disabilities can navigate independently in complex environments such as college campuses and large cities	The results indicated that the augmented reality navigation application was functionally the most effective condition
**Peñaloza-Salazar et al. (2016)** *Cognitive mechanisms underlying Armoni: A computer-assisted cognitive training programme for individuals with intellectual disabilities* [42]	59	19–56	46% female 54% male	Etiology of ID was heterogeneous and, in many cases, unknown	Mild, moderate, or severe	Determine the cognitive mechanisms underlying 16 activities included in Armoni, a computerized cognitive training program for individuals with ID, in order to validate its use with this population	Participants were given the task instructions and were encouraged to complete the activity; cognitive testing was conducted in two sessions, with the original test instructions being slightly modified to facilitate comprehension	Most of these activities showed a significant correlation with visuoconstruction comprehension and naming ability and presented a varying number of significant relationships with the remaining cognitive domains
**Cano, A.R., Fernández-Manjón, B. and García-Tejedor, Á.J. (2018)** *Using game learning analytics for validating the design of a learning game for adults with intellectual disabilities* [43]	51	19–41	-	58,8% Down syndrome 41.2% intellectual disabilities and certain types of autism spectrum disorder	Mild cognitive disability or certain types of autism spectrum disorder	The use of a serious game to train students with intellectual disabilities in traveling on the subway as a complement to traditional training	Used standards-based game learning analytics techniques to collect and analyze learning data both off-line and in near-real-time while the users were playing the video game; analyzes and assesses the evidence data collected using analytics during the game sessions, such as time completing tasks, inactivity times, or the number of correct/incorrect stations while traveling	Differences between high-functioning and medium-functioning users were found; the fact that almost all of the students completed at least one route without mistakes, the general improvement through sessions, and the low mistake ratio were good indicators of the appropriateness of the game design
**Cano, García-Tejedor, Alonso-Fernández and Fernández-Manjón, B. (2019).** *Game Analytics Evidence-Based Evaluation of a Learning Game for Intellectual Disabled Users* [44]	51	19–41	-	58.8% Down syndrome 41.2% intellectual disabilities and certain types of autism spectrum disorder	Mild cognitive disability or certain types of autism spectrum disorder	The use of a serious game to train students with intellectual disabilities in traveling on the subway as a complement to traditional training	Evidence-based approach for evaluating the game design of Downtown, A Subway Adventure, a game created to improve independent living in users with ID	The proposed evidence-based approach using game analytics information is an effective way to evaluate both the game design and the implementation, especially in situations where other types of evaluations that require users’ involvement are limited
**Derks et al. (2019)** *Effectiveness of the serious game ‘You & I’ in changing mentalizing abilities of adults with mild to borderline intellectual disabilities: a parallel superiority randomized controlled trial* [45]	172 adults with MBID,	18 years or older	-	Adults with mild to borderline intellectual disabilities and autism spectrum quotient	IQ ranging between 50 and 85 adults with mild to borderline intellectual disabilities	Examine the effectiveness of the serious game “You & I” in changing mentalizing abilities and stress regulation	Randomized controlled trial (RCT) with a baseline, a post test after 4 weeks, and a follow-up assessment after 6–8 weeks	The serious game “You & I” aims to improve mentalizing abilities in adults with mild to borderline intellectual disabilities, which is expected to lead to improved regulation of stress in social relationships; the serious game can be readily implemented on a broad scale in the care organizations for people with intellectual disabilities, thanks to the low cost of deployment
**Lara et al. (2019)** *A Serious Videogame to Support Emotional Awareness of people with Down Syndrome* [46]	7	31–36	-	Adults with Down syndrome	Mild cognitive disability	Creation of the initial design characteristics that emerged from the contextual study	During the videogame testing sessions, it was observed that videogames using touch-based interaction are a valuable tool for supporting skills of people with Down syndrome since they provide a natural interaction for them, and they could also help them practice fine motor skills	The results from the contextual study indicate that people with Down syndrome have different emotional problems regardless of their age or level of functionality
**Benda et al. (2019)** *Practical Education Of Adults With Intellectual Disabilities Using A Web Course* [47]	10	37–62	-	5 perinatal hypoxia–ischemia 5 Down syndrome	5 mild, 4 moderate 1 severe	Verify whether a web course could be an effective educational tool for people with intellectual disabilities	Validated observation T-tests were used to compare means	A web course, with specific content that consists of video or animation, combined with the use of pictograms for confident navigation, can be used by people with intellectual disabilities with good results; repetition using the web course was a better method than simple verbal repetition only
**Bridges et al. (2020)** *Augmented Reality: Teaching Daily Living Skills to Adults With Intellectual Disabilities* [48]	3	-	2 male 1 female	1 William syndrome, 1 Down syndrome 1 1 attention deficit hyperactivity disorder and atypical behaviors of pervasive developmental disorder	IQ-63, IQ-52	The design was used to examine the efficacy of an augmented reality intervention for teaching daily living skills	Multiple-baseline designs were used for the intervention. One participant received the intervention, while the others remained in the baseline condition; material: HP reveal and the social validity questionnaire determine the efficacy of an intervention when the participants are unlikely to return to baseline levels after being introduced to the intervention	The intervention was effective for increasing independence among all participants; the use of augmented reality is an effective tool for teaching daily living skills to adults with ID; using augmented reality) as an intervention is not only effective, but practical for use in natural environments
**Michalsen et al. (2020)** *Physical Activity With Tailored mHealth Support for Individuals With Intellectual Disabilities: Protocol for a Randomized Controlled Trial* [49]	60	18–60	-	Adults with low activity and mild ID	Mild ID	Design was used to measure physical, memory, and planning functions	The current study had a randomized controlled clinical design with assessments at baseline, 3 months, and 6 months; participants will receive either the tailored mHealth-supported PA program or standard care with access to the mHealth support system once the trial was complete; family and care staff will be involved in the program for support and follow-up	The Self-Efficacy/Social Support for Activity for Persons with Intellectual Disability scale [35] is a questionnaire consisting of 4 subscales: one subscale measures self-efficacy for overcoming barriers to leisure PA; the last 3 subscales measure social support for leisure activity from family members, care staff, and friends for individuals with IDs; the scale has been validated for self-reporting from individuals with mild to moderate IDs and for use by proxy respondents
**Gibson et al. (2020)** *Designing Clinical AAC Tablet Applications with Adults who have Mild Intellectual Disabilities* [50]	10	18–60	5 males and 5 females	Adults with mild ID	Mild ID	Augmentative and alternative communication (AAC) technologies	Participation in four tasks: focus group, image board, paper prototype, and prototype evaluation, with these tasks previously being adjusted (with the help of experts in ID) to cater to the accessibility needs of the participants	Such technologies can promote communication between general practitioners and patients with mild ID by extracting symptoms in advance of the consultation via an accessible questionnaire; the application can support people with ID in identifying and accessing healthcare services

## Data Availability

The data used in the study can be accessed by direct reference from the references section.

## Review Limitations and Future Research

It is important to note that this review only selected studies published in English. Studies in other languages were not considered at this stage of the research. Most of the serious game studies are related to children and adolescents with ID and are centered on education and promoting cognitive development. From the review, it was evidenced that few studies were focused on adults with ID and especially on EFs in order to promote autonomy in daily living. Moreover, few of the studies included the analysis of effectiveness of the serious games and were only focused on their design and description. Improvement of the technology resources for adult and senior categories of people with intellectual disabilities is needed for future use.
